# Debunking myths in medical education: The science of refutation

**DOI:** 10.1111/medu.14028

**Published:** 2019-12-17

**Authors:** Anique B H de Bruin

**Affiliations:** ^1^ School of Health Professions Education Maastricht University Maastricht the Netherlands

## Abstract

de Bruin argues that efforts to eradicate erroneous assumptions students and teachers hold about learning requires refutation, a process that combines both correct scientific knowledge and rejection of misconceptions.

Of all the many individual differences studied in education research, one particular factor consistently affects the chance that an individual will learn new information and explains more variance in learning than many other factors combined: the student's level of prior knowledge. More than motivation, socioeconomic status, self‐regulation skills and gender, prior knowledge provides a basis for new learning by allowing the connection of new information to knowledge that is already stored in existing schemata.^1^ Students with prior topic knowledge are more likely to remember new information,^2^ can handle greater complexity of information,^3^ and may need a different instructional format compared with those with low prior knowledge.^4^

… the human mind is loyal to what it has known and used for a longer period, even when confronted with the incorrectness of that knowledge

But what happens when a learner's prior knowledge is incorrect or when novel scientific insights require the updating of existing knowledge? That is when things get rough. When prior knowledge is incorrect, it is more difficult to gain the correct knowledge than when no prior knowledge is available.^5^ It is particularly hard for learners to let go of incorrect information. Prior knowledge is resistant to change because the human mind is loyal to what it has known and used for a longer period, even when confronted with the incorrectness of that knowledge. Living in an era of information explosion, in which the sources of information vary in reliability, makes for a potentially toxic context in which knowledge is gained easily, but is hard to reject or replace once it has been internalised.

Living in an era of information explosion makes for a potentially toxic context in which knowledge is gained easily, but is hard to reject or replace …

The papers by Rudland et al,^6^ Masters^7^ and Molloy et al^8^ on our often‐erroneous knowledge about the effects of stress, our use of Dale's pyramid of learning, and the myths of feedback all underline how pervasive incorrect knowledge is and how it continues to inform our teaching practices well after it has been debunked. Add to this the omnipresent myth of learning styles (ie that learners have an optimal mode of processing information such that, for example, ‘visual learners’ learn more easily from visually presented material and ‘aural learners’ learn more easily from auditory information^9^ amongst other things), and we see ourselves faced with a quest to explicitly debunk myths in our teaching. Medical education is not organic chemistry or quantum physics, in which universal laws apply and are maintained over centuries. Instead, medical education is more typically characterised by limited evidence, varying evidence or, most commonly, a lack of evidence regarding the assumptions we hold about learning. Some of our intuitions will be correct, but the omnipresence of learning myths calls for design, testing and the implementation of teaching strategies that specifically address the issue of how to maximise the rejection of incorrect knowledge and encourage its replacement with correct knowledge in such a way that learners will use it in practice.

… learners should engage with the scientifically correct information extensively and should be given opportunities to explore their own incorrect ideas and to attempt to understand the errors in their thinking

Studies in education psychology have addressed this issue through the consideration of myth debunking for about four decades and the insights this research has gained have potential to inform research and teaching in medical education. Although these studies were often performed in younger learners (eg adolescents) and mostly related to learning concepts in the natural sciences, their findings have revealed several generalities of relevance. First and foremost, changing learners’ incorrect knowledge is an issue of substantive reorganisation of knowledge, not a matter of ‘telling students how it is.’ Just as debiasing is insufficient to improve clinical reasoning,^10^ providing the correct knowledge alone will not produce the reorganisation of knowledge that is ultimately required. The task of overcoming incorrect knowledge is, therefore, often referred to as an issue of enabling ‘conceptual change.’ To do so, learners should engage with the scientifically correct information extensively and should be given opportunities to explore their own incorrect ideas and to attempt to understand the errors in their thinking.^11^ Often, scientific explanations are counterintuitive to the naïve ideas and experiences of the learner (as in the case of the learning style myth), which makes it particularly necessary to elaborate on the reasoning behind the scientific explanation and have learners actively contrast it with naïve conceptions. The correct information and the misconception should be co‐activated in working memory in order for them to be changed.^12^ One promising approach to doing this is to use ‘refutations.’ Refutation texts combine an explanation of correct scientific knowledge with an explicit rejection of learners’ misconceptions. This is often in the form of: ‘Some people think X. However, this is not true/there is no evidence for this assumption. Instead, there is evidence for Y’ (followed by a detailed explanation of Y). The incorporation of the refutation encourages active contrasting with the correct scientific information and explanation and thereby increases the chance that misconceptions will be rejected and the correct information internalised.^13^ Refutation texts have also been observed to make students interact more actively with the learning material, to improve the accuracy of meta‐comprehension^14^ (ie learners’ self‐assessments of their understanding), and to increase the transfer of newly gained knowledge to other contexts.^15^

Refutation texts … make students interact more actively with the learning material, to improve the accuracy of meta‐comprehension …

Rather than simply being useful for facilitating conceptual change in students, such findings suggest ways in which the presentation of our scientific writing might better facilitate conceptual change in our readers. For example, perhaps the explicit message refuting the evidence for Dale's pyramid of learning, as stated by Masters^7^ at the end of his abstract, would be made even more influential if it started with: ‘Some people think that [learners remember 10% of what they hear from lectures]. However, there is no evidence for this.’ It is for reasons such as this that explicit examination of our field's mythology is so important. If the erroneous assumptions students and teachers hold about learning must be tackled explicitly and extensively through refutations, then so too is it likely that our research activities must similarly begin to incorporate refutations as a means through which to change our collective conceptualisations. Moving forward in our field depends on acknowledging that many of our collective conceptualisations are not supported by evidence, and that several will be rejected in the near future. Becoming aware of students’ prior knowledge and the possible errors it contains is a prerequisite step to optimising the effect our teaching has on students’ knowledge adaptation and construction; it is similarly likely to be a prerequisite step towards doing the same for our field.

Becoming aware of students’ prior knowledge and the possible errors it contains is a prerequisite step to optimising the effect our teaching has on students …
